# How Women Were Affected by the Tsunami: A Perspective from Oxfam

**DOI:** 10.1371/journal.pmed.0020178

**Published:** 2005-06-28

**Authors:** Rhona MacDonald

## Abstract

When natural disasters hit poverty-stricken areas, women are more likely to be affected than men. MacDonald discusses the reasons for, and implications of, this gender disparity.

**Figure:**
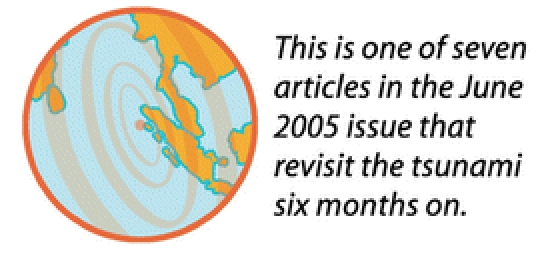


In Oxfam's experience, disasters are profoundly discriminatory, even those that are “natural” rather than man-made. Factors that were present before a disaster, such as poor social conditions, mean that some people in the disaster zone will be more affected than others.

People living in poverty are much more vulnerable to the effects of natural disasters. As women account for 70% of the 1.3 billion people worldwide living in extreme poverty (less than $1 a day) (see http://www.oxfam.org.uk/what_we_do/issues/gender/introduction.htm), it follows that when natural disasters hit poverty-stricken areas, women are more likely to be affected than men.

## Disproportionate Death Toll in Women

The tsunami decimated Southeast Asia on 26 December 2004, killing more than 220,000 people in 12 countries and leaving 1.6 million people homeless. According to a survey recently carried out by Oxfam, four times as many women than men were killed in the tsunami-affected areas of Indonesia, Sri Lanka, and India [1]. Some of the reasons for this are similar across these countries: women died because they stayed behind to look for their children and other relatives. Women in these areas often can't swim or climb trees, which meant that they couldn't escape.

Some cultural differences between men and women also contributed to the disproportionate death toll. Although traditionally many women go out to work in Aceh (Indonesia), the tsunami hit on a Sunday morning when they were at home and the men were out on errands and so away from the seafront. Women in India play a major role in fishing and were waiting on the shore for the fishermen to bring in the catch when the wave struck. In Batticaloa District of Sri Lanka, the tsunami hit at the time when women who lived on the east coast usually took their baths in the sea [1].

## Gender-Specific Problems

**Figure 1:**
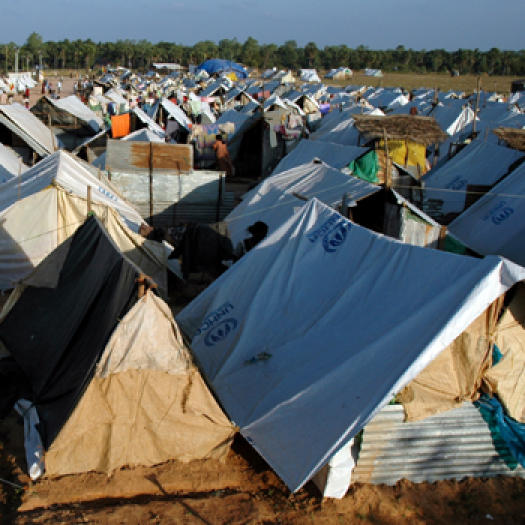
Camps and Resettlement Sites Are Overcrowded (Photo: Howard Davies, Oxfam)

The overcrowded camps (Figure 1) and resettlement sites for people who have been made homeless and the imbalanced male-to-female survival ratio have resulted in several gender-specific problems. For example, women in the camps are often verbally and physically harassed by men and are at risk of being sexually abused.

It is also more likely that women will be pressured into marrying earlier than in the past and into having more children closer together, with significant implications for their education, livelihoods, and reproductive health. It is also more difficult for women to access money and emergency supplies because in some areas only men are recognised as head of the household, which has meant that women have been unable to collect entitled relief cash and goods [1].

All of the above problems are an escalation of the reality that women faced before the tsunami. For example, Oxfam has been working on gender issues in the Batticaloa District of Sri Lanka for four years. According to Shanthi Sivasanan, Oxfam programme assistant in the area: “Before the tsunami, there were some very serious issues regarding the level of violence against women in this area, and we need to continue considering this during our tsunami response. Our ongoing development programme was offering counselling to families and people affected by violence (whether through war or domestic violence, etc.), training for counsellors, paralegal training, and the protection of children.”

“From 2002 to 2004, we were aware of 400 cases of very critical domestic violence. We found that out of these 400, 150 women were physically injured in their own home *every* day. And 48 suffered mental harassment. Often, this was all due to stress and pressure in the home because of dowry payments, political issues, and poverty.”

The tight culture means that if women experience violence, they will never talk about it. Pathimalar, a survivor of the tsunami currently living in Vattavan Camp in Batticaloa District, said: “Many women, like me, are uneducated. We have a saying in Tamil which says, ‘Even if our husband is a stone, we worship him.’ We don't like to talk about harassments or what happens between man and wife. If something happens and we start to cry, or shout too much, we think it might create further problems and he starts to fight with us again.” Pathimalar explained how women deal with this situation: “Silently and patiently, we cry within us.”

## Meeting Women's Needs in the Emergency and Recovery Phase

Oxfam's programme strategy is to assess the needs of women and men in all of its work. So in the relief phase, in addition to supplying shelter, mosquito nets, clean water, food, and cooking utensils, Oxfam distributed public-health packages that included toiletries and underclothes for women. Oxfam also consulted women in the camps about emergency water and sanitation interventions and also asked them about their specific needs. This has resulted in building well-lit toilets, where the women feel safe.

Immediately after the tsunami, it was very difficult for the survivors who had been made homeless to follow their domestic hygiene practices. Now that Oxfam has built clean latrines and distributed soap, health promoters have been reinforcing the basic rules of hygiene by using visual aids and drama.

According to Jastinahilari Ignatius and Jesudhas Yeogaraja, Oxfam health supervisors in Vattavan Camp, gender issues mean that they have to have separate groups for men and women when teaching health promotion: “There are some difficult gender issues here, and some of the women don't like to speak out in front of men, so we had separate groups for men and for women, with the result that all of the participants talked more than they would have done in a mixed group.”

Pregnant women living in the camps have a very uncomfortable time, according to V. Uijayaluxumi, a field health officer with Oxfam's partner, Sarvodaya, working in Vattavan Camp: “Many of the pregnant women have been really psychologically affected by the tsunami. They were scared for themselves and their families, but especially for their unborn children. Now they are [here] in camps and living in tents where it's hot, noisy, and away from familiar surroundings. There's just dust and tents here.”

“It is traditional here for women to use many herbs and remedies before and after giving birth, but they can't get these while they are in the camps. They use things like honey, tree bark, and leaves, etc., and there is one powder that is supposed to be good for the stomach and helps with feeding and sleeping.”

She also reported some good news: “One baby has already been born in this camp and is doing well, but there are nine more due before July.”

In the reconstruction work, Oxfam aims to ensure equal wages for equal work, regardless of gender. For example, in Culladore, India, Oxfam worked with the district administration to ensure that men and women were paid equally for the same work. Men, who were previously paid higher wages, resisted this initially, but Oxfam staff and partners talked to the community, and everyone eventually agreed that equal pay was the right thing to do [1].

There are some positive examples of governments recognizing the important role of women after the tsunami. For example, just after the tsunami, the governments of Tamil Nadu and Kerala (in India) implemented an initiative to post women fire officers, police officers, and doctors in the camps and affected villages. This helped to prevent violence against women and provided women survivors with a safer environment [1].

## Conclusion: Gender Equality and Global Poverty

Oxfam bases its work on the common understanding that gender equality is the key to overcoming poverty and suffering (see http://www.oxfam.org.uk/what_we_do/issues/gender/policy.htm). The special needs of women—whether or not these needs are related to one of the biggest natural disasters the world has ever seen—must be urgently addressed by the international community. After all, world leaders agreed to a Millennium Development Goal to promote gender equality and empower women [2]. Those of us in the international health and development community must keep reminding these leaders of their commitment, and keep campaigning for some progress.
